# Tuning TiFe_1–*x*_Ni_*x*_ Hydride Thermodynamics through Compositional Tailoring

**DOI:** 10.1021/acsaem.4c02625

**Published:** 2025-02-06

**Authors:** Evans Pericoli, Viola Ferretti, Dario Verna, Luca Pasquini

**Affiliations:** Department of Physics and Astronomy A. Righi, University of Bologna, Bologna 40127, Italy

**Keywords:** hydrogen storage, metal hydrides, compositional tailoring, structural properties, enthalpy−entropy compensation, high-pressure differential scanning calorimetry

## Abstract

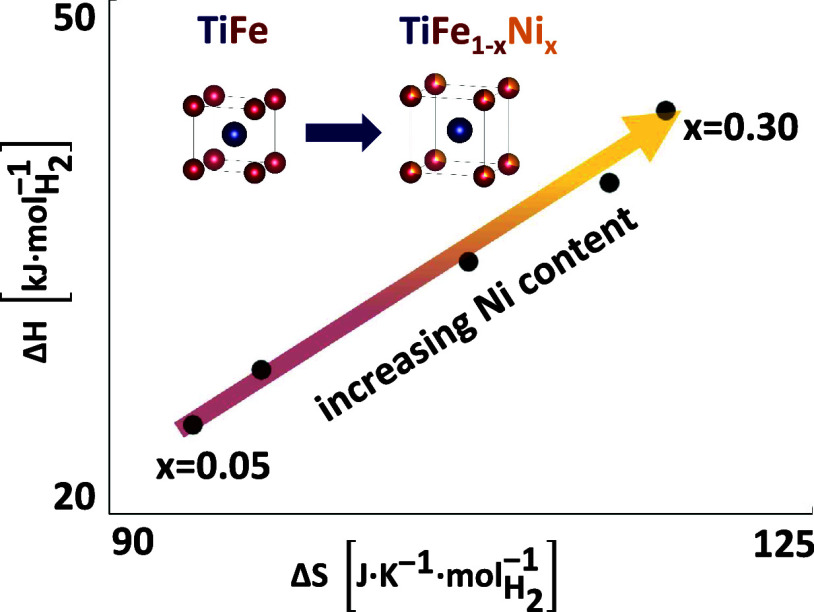

In this study, we investigate how structural modifications induced by Fe substitution with Ni in the TiFe intermetallic alloy affect the thermodynamics of hydride formation and decomposition. The primary goal of substituting Fe with Ni was to reduce the plateau pressure of TiFe, a crucial parameter for reversible solid-state hydrogen storage applications under near-ambient conditions (below 150 °C and 50 bar). Alloy compositions TiFe_1–*x*_Ni_*x*_ with *x* ≤ 0.30 were synthesized by arc melting. The structural and morphological properties were characterized using powder X-ray diffraction and scanning electron microscopy with energy-dispersive X-ray spectroscopy. The thermodynamic properties were investigated through volumetric measurements using a Sieverts’ apparatus and calorimetric analysis with a high-pressure differential scanning calorimeter. We show that Ni incorporation effectively lowers the plateau pressure, stabilizing the hydride thermodynamics due to a more negative enthalpy of hydride formation. Moreover, the entropy of hydride formation increases with the Ni content, resulting in a linear correlation between the enthalpy and entropy values determined at different compositions. The enthalpy–entropy compensation effect was analyzed to determine whether it arises from statistical artifacts or is genuine to the system, as our findings suggest.

## 1 Introduction

Hydrogen is progressively recognized as a critical component in transitioning to a low-carbon energy future, as highlighted by the International Energy Agency (IEA) roadmap.^1,2^ It is a versatile energy carrier, important especially in hard-to-abate sectors like heavy industry, aviation, and long-distance transportation, which makes it a key element in the future energy landscape.^3^

However, the general adoption of hydrogen relies on overcoming several technical and economic limitations, with storage representing one of the most substantial.^4^ Current storage methods, including high-pressure gas tanks, cryogenic liquid storage, and solid-state metal hydrides, have constraints in cost, efficiency, and energy density.^5^

The need for a practical storage system that meets operational ambient conditions, such as when coupled with electrolyzers and fuel cells, has renewed interest in metal hydride chemical storage. Among materials considered for solid-state hydrogen storage, TiFe-based alloys are particularly promising for stationary applications due to their balanced combination of low cost, low environmental impact, reversibility, and favorable hydrogen sorption kinetics.^6−8^ The cost of the materials can be even lower if iron and titanium are recovered from other industrial processes.^9^

These alloys operate effectively at moderate temperatures and pressures below 100 bar and 100 °C. The maximum reported gravimetric and volumetric densities at 25 °C (70 bar) are 1.87 wt% and 105 kg m^–3^, respectively.^10^ These properties make them suitable for practical applications under ambient conditions.^6^

Reilly and Wiswall^11^ were the first to describe the properties of iron–titanium hydrides. They reported the existence of two ternary hydrides: FeTiH_≈1_ (β phase) and FeTiH_≈2_ (γ phase). They estimated the critical temperature for the β–γ transition to be close to 55 °C. The transition between these two phases is almost continuous in the temperature range that we have investigated, represented by an almost linear variation of concentration with pressure. For this work, we have decided to limit the H_2_ pressure to 30 bar, the maximum output pressure of most commercial electrolysis systems.^12^

TiFe exists in a narrow compositional range, from 49.5 to 52.5 at% Ti at 1000 °C, crystallizing in the CsCl-type structure (space group *Pm*3̅*m*).^13^ Secondary phases emerge outside this compositional interval, on both the Ti-rich and Fe-rich sides, significantly altering the hydride sorption properties. As an example, Ti_4_Fe_2_O-type phases (cubic phase, space group *Fd*3̅*m*)^14^ form on the Ti-rich side when oxygen contamination occurs. In a recent work, Liu et al.^15^ have investigated the role of these suboxides as gateways for hydrogen absorption and easier activation. While these phases can absorb hydrogen, they show a reduced total capacity compared to pure TiFe. Zavaliy et al. reported a maximum gravimetric density for Ti_4_Fe_2_O_0.25_ of 1.58 wt% at RT, 1.5 bar.^14^ The high stability of these hydrides, which show decomposition temperatures^14^ above 200 °C, can therefore lead to a total reduction of the maximum reversible storage capacity.

Significant challenges must be addressed to transition these compounds from laboratory-scale prototypes to industrial-scale operating systems. These include harsh activation conditions—typically a 400 °C heat treatment under a pressurized hydrogen atmosphere^6,16^ —and a strong dependence of hydrogen storage performance—total capacity, pressure–temperature conditions, and hydrogen uptake/release rate—on precise stoichiometric composition, including the presence of secondary phases, substitutions, or impurities.^13^

One way to improve the thermodynamic and kinetic properties of these alloys while addressing the activation issue concerns stoichiometric tailoring, which involves adjusting the ratio of titanium (Ti) to iron (Fe) and incorporating substitutional elements, such as V, Cr, Co, Ni, Mn, or Zr.^6^ These substitutions can alter the phase stability and sorption behavior by affecting the microstructure and electronic structure of the alloy, potentially improving activation conditions,^16^ cyclability,^17^ and thermodynamic and kinetic performances.^18^ However, this may result in a reduction of the total capacity of the compound,^13^ often caused by the formation of precipitates and secondary phases, which do not react with hydrogen or form stable hydrides.

This study aims to investigate the effect of Ni partial substitution of Fe in the TiFe_1–*x*_Ni_*x*_ system, focusing on its thermodynamic properties.

Nickel is now classified as a critical raw material by the European Union,^19^ meeting the conditions to be considered a strategic raw material, as its importance in the strategic technologies, related to the energy transition and defense, grows. Nevertheless, its supply chain remains solid, and its adoption as a substituent is a viable solution given its low toxicity and high recyclability.^20^

In substituting Fe with Ni, Mintz et al.^21^ reported an increased stability of the monohydride phase and a decreased stability of the dihydride phase. They also reported a significant decrease in the hysteresis. More recent studies have also shown that partially substituting Fe with Ni in the TiFe_1–*x*_Ni_*x*_ system can lower the plateau pressure. This adjustment potentially leads to easier activation^16^ and enhanced hydride stability.^18,22,23^ Interestingly, the relationship between Ni substitution, unit cell expansion, and some aspects of the resulting thermodynamics has not yet been fully explored.^16^ For instance, enthalpy–entropy compensation (EEC) is often observed in chemical and biological processes and manifests as a linear correlation between the enthalpy and the entropy of reaction.^24^ The existence of the EEC has an impact on the tailoring of thermodynamic properties via elemental substitution. In this work, we apply a recently developed statistical test to assess whether the observed correlation between the enthalpy and entropy with varying nickel contents is a genuine effect.

By systematically varying the Ni content in the range *x* ≤ 0.30, we seek to understand how unit cell expansion resulting from Ni incorporation impacts the enthalpy and entropy of hydride formation and decomposition, which in turn affects the operational temperature–pressure conditions for hydrogen sorption.

Given that Ni has a slightly smaller atomic radius compared to Fe (*r*_Ni_ = 1.24 Å while *r*_Fe_ = 1.26 Å, where the atomic radius is defined as half the internuclear distance between the two nearest neighbors in the pure metal)^25,26^ but forms a weaker Ti–Ni bond^27^ with a larger bond length compared to Ti–Fe, its substitution is expected to expand the unit cell, facilitating hydrogen accommodation in the interstitial sites.^18^

To characterize the thermodynamic properties of TiFe_1–*x*_Ni_*x*_ hydrides, we combine structural, microstructural, and compositional analyses with volumetric and calorimetric measurements using a Sieverts’ apparatus and a high-pressure differential scanning calorimeter (HP-DSC). The key advantage of coupling these techniques lies in the ability to analyze multiple compounds more rapidly, gaining valuable insights into the dependence of hydride thermodynamics on structural parameters and composition.

## 2 Experimental Section

### 2.1 Sample Synthesis

A total of six compounds were synthesized, ranging from equiatomic TiFe (sample name: Ni_0_) to TiFe_0.70_Ni_0.30_ (sample name: Ni_30_), as reported in Table 1. The alloys were prepared by arc melting in an argon atmosphere on a water-cooled copper plate, using an Edmund Bühler compact arc melter. Each sample was melted three times on both sides to ensure structural homogeneity. Bulky grains of Ti (Thermo Scientific, 1–2 mm, 99.9%), Fe (Thermo Scientific, 1–2 mm, 99.98%), and Ni wires (Thermo Scientific, 0.5 mm, 99.98%) were mixed to achieve the desired compositions. The average amount of material produced was around 1.1 g with a weight loss after melting below 1%. For each composition, two samples were synthesized: one underwent a metallographic procedure, including high-precision cutting and cross-sectional polishing in preparation for microanalysis. The other was first crushed in a mortar and then ball milled for 10 min in a N_2_ atmosphere using a high-energy Spex CertiPrep 8000 mixer/mill, equipped with stainless-steel balls at a ball-to-powder ratio of 10:1. A total of two light balls (1 g each, 6.2 mm diameter) and one larger ball (8 g, 12.7 mm diameter) were used for the mechanical treatment, and the typical sample size was 1 g.

**Table 1:** Sample Names, Stoichiometric and Nominal Compositions (at%), and Elemental Abundance (at%) Obtained from EDS Microanalysis^a^

**sample**		**nominal composition** [at%]	**Ti** [at%]	**Fe** [at%]	**Ni** [at%]
Ni_0_	TiFe	Ti_50_Fe_50_	51.2(4)	48.8(4)	0
Ni_5_	TiFe_0.95_Ni_0.05_	Ti_50_Fe_47.5_Ni_2.5_	50.8(3)	46.6(3)	2.7(1)
Ni_10_	TiFe_0.90_Ni_0.10_	Ti_50_Fe_45_Ni_5_	50.9(1)	44.1(1)	4.9(1)
Ni_15_	TiFe_0.85_Ni_0.15_	Ti_50_Fe_42.5_Ni_7.5_	52.1(3)	41.0(2)	6.9(3)
Ni_20_	TiFe_0.80_Ni_0.20_	Ti_50_Fe_40_Ni_10_	51.5(2)	38.9(2)	9.6(1)
Ni_30_	TiFe_0.70_Ni_0.30_	Ti_50_Fe_35_Ni_15_	51.2(3)	34.0(3)	14.8(2)

a The numbers in parentheses represent the standard deviation in units of the last significant digit.

This mechanical treatment reduced the preferential orientation observed in the X-ray diffraction profiles and facilitated the activation process. Milling for a short period of time effectively cracks the external passivating layer, exposing fresh surfaces ready to interact with hydrogen.^28^ The obtained powder was used for all of the subsequent analyses, as described below.

### 2.2 Structural and Compositional Characterization

Powder X-ray diffraction (XRD) profiles were collected at ambient conditions using an X’Pert Pro Panalytical Bragg–Brentano diffractometer equipped with the X’Celerator multichannel solid-state detector and a source of Cu Kα radiation. The 2θ range of 28–115° with a step size of 0.017° and a time per step of 55 s was used for all samples. The diffraction patterns were analyzed via Rietveld refinement^29^ using MAUD software.^30^ The instrumental function was determined from the Rietveld refinement of a reference LaB_6_ powder.

Compositional analyses on the polished cross sections were conducted using a LEICA Cambridge Stereoscan 360 scanning electron microscope (SEM) operated at 20 kV and equipped with an Oxford Instruments 7060 detector for energy-dispersive X-ray microanalysis (EDS). The calibration of the EDS was carried out by using a cobalt reference according to standard practice. INCA software was used to extract elemental compositions from the EDS data. Each section was analyzed by local mapping on 10 spots to check homogeneity; the standard deviation was used to determine the uncertainty in the composition.

### 2.3 Thermodynamic Characterization

Pressure–composition isotherms (PCIs) were measured using a custom Sieverts’ type apparatus equipped with an MKS Baratron 750D13PCD2GA pressure sensor, with a 1% accuracy on the readings. The typical mass of the sample in a powder form was 1 g. The activation procedure involved an isothermal treatment at 400 °C under 40 bar H_2_. Then, eight absorption (under 40 bar H_2_) and desorption (in dynamic vacuum) cycles at 100 °C were carried out to ensure complete activation. The PCI curves were measured over the pressure range from 0.1 to 30 bar, which is relevant for coupling electrolyzers that deliver H_2_ below 30 bar. This means that only the first plateau of the TiFeH system, corresponding to the formation and decomposition of the monohydride phase, was investigated, while the transition to dihydride was not completed. Correspondingly, the temperature range spanned from 30 to 170 °C. After each PCI cycle, samples were exposed to dynamic vacuum for 20 min and then heated up to higher temperatures.

The midplateau pressure values were used to determine the absorption/desorption equilibrium points, from which the enthalpy and entropy of formation and decomposition were extracted by Van ’t Hoff analysis of the pressure–temperature data. The separate values obtained for absorption and desorption are reported in Table S2. The geometric mean of the absorption/desorption pressures was used to determine the mean enthalpy Δ*H*_PCI_ and entropy Δ*S*_PCI_ reported in Table 3.

Calorimetric measurements were conducted by using a TA Instruments HP-DSC Q10 system. The instrument was calibrated with indium, and a baseline correction was applied by using empty sample and reference pans. The activation procedure consisted of an initial thermal treatment similar to the one described for Sieverts’ apparatus and was completed by performing 10 absorption/desorption ramps under 40 bar H_2_. Finally, heating/cooling ramps from 50 to 350 °C with a scan rate of 5 °C/min were collected under various H_2_ pressures in the 5–40 bar range. To obtain the enthalpy and entropy of the sorption process from HP-DSC data, we identified the temperature value that divides the integral area in half for each ramp, resulting in T_ABS_ and T_DES_. This procedure enabled a more reliable determination of the equilibrium temperature and pressure compared to the straightforward use of peak temperature, typically influenced by the process kinetics, as the onset temperature, which can be, in addition, challenging to identify. The Van ‘t Hoff analysis was then carried out using the average value of the former temperatures plotted against the mean value of the corresponding pressures.

## 3 Results

### 3.1 Structural and Compositional Analysis

Table 1 lists the synthesized compositions and the elemental abundances obtained from EDS microanalysis. The Ti content in the samples ranges from 50.8 to 52.1 at%. While the Ti at% is slightly higher than the nominal composition and the Fe at% is correspondingly lower, neither Ti-rich particles nor other secondary phases were detected by SEM observations of the polished cross sections (Figure S1). This suggests a homogeneous TiFe_1–*x*_Ni_*x*_ matrix for all samples.

**Figure 1:**
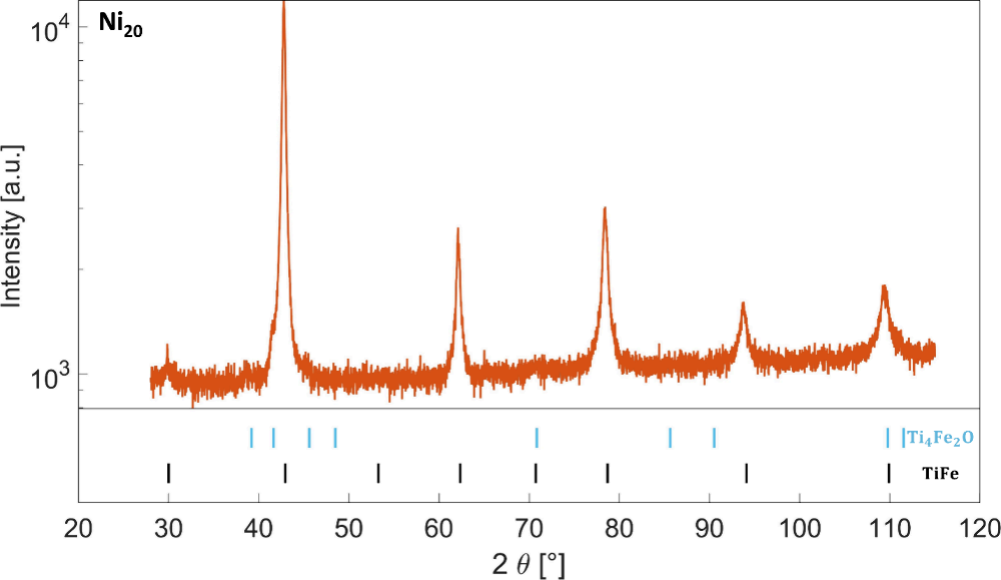
XRD profile of sample Ni_20_. The positions of the Bragg reflections of TiFe and Ti_4_Fe_2_O are indicated.

Figure 1 shows the XRD profile of Ni_20_; similar results were obtained on the other samples, as displayed in Figure S2. The main peaks are associated with the BCC B2 structure (space group *Pm*3̅*m*), with a superlattice reflection at about 30°. In all samples, two weak peaks at about 39 and 42° are detected, which correspond to the most intense reflections of Ti_4_Fe_2_O, one of the most common suboxides that form in these systems.^6,10,13^ The latter phase shows a maximum abundance of 5 wt% in Ni_10_ and no apparent correlation with the nickel content. The absence of the suboxide particles in the SEM and EDS analyses of polished cross sections suggests that the secondary phase is either preferentially located at the external surface of the samples or present in nanosized precipitates.

It is reasonable to assume that the excess Ti detected by EDS, which may have been caused by stronger Fe and Ni evaporation during melting, either occupies the Fe sites in TiFe or is consumed by Ti_4_Fe_2_O formation. Thus, XRD profiles have been fitted using a homogeneous TiFe phase with the occupancy determined through EDS analysis, assuming that both the excess Ti (≤2 at% in all samples) and the substituent Ni occupy the Fe sites. A second iteration was then carried out, correcting the excess Ti in TiFe based on the determination of the Ti_4_Fe_2_O content; the results showed only minimal changes, often within the experimental uncertainties, as expected given the small amount of both suboxide and excess Ti. To further test the sensitivity of the Rietveld analysis to the compositional details, we compared the results to those obtained using the nominal compositions without excess Ti and found no difference within the experimental uncertainties.

**Figure 2:**
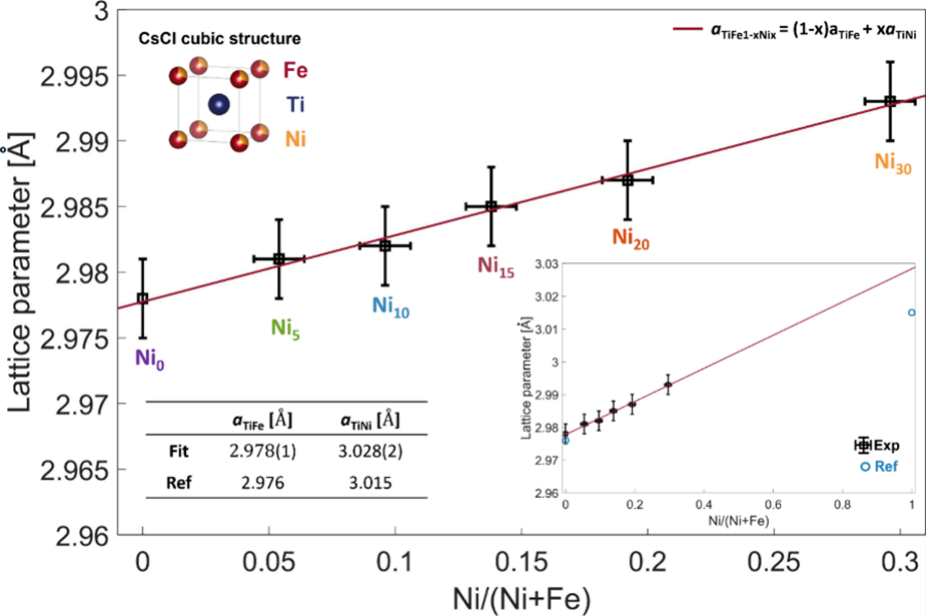
Enlargement of the lattice parameter as a function of the Ni/(Ni+Fe) ratio (black squares) closely follows Vegard’s law in the form  = (1 −*x*)*a*_TiFe_ + *xa*_TiNi_ (red line). Based on this trend, the lattice parameters of TiFe and TiNi can be extracted, showing excellent agreement for TiFe values and a slight discrepancy for TiNi. The top inset illustrates the atomic arrangement of Ti and Fe atoms in a BCC CsCl-like structure with Ni substitutions of Fe sites. The bottom inset shows the comparison between experimental and literature values (blue circles).

The lattice parameter (Table 2) of the equiatomic TiFe composition Ni_0_ (2.978(3) Å) is compatible with values reported in the literature (2.976 Å).^6,10,13^ Good agreement with the literature is also observed in Ni-substituted compounds.^17,21,31^ Notably, increasing the Ni content results in monotonous lattice parameter enlargement up to 2.993 Å in sample Ni_30_ (Figure 2). The linear fit in the explored regime (the red line and inset in Figure 2) yields an extrapolated lattice parameter *a*_TiNi_ = 3.028(2) Å for Ni/(Ni + Fe) = 1. This value is larger than the experimental one (3.015 Å) for TiNi,^27^ suggesting a nonideality of the TiFe_1–*x*_Ni_*x*_ solid solution. The change in the lattice parameter does not correlate with the amount of excess Ti, confirming that Ni substitution for Fe is the dominant cause of unit cell expansion.

**Table 2:** Lattice Parameter *a* of TiFe_1–*x*_Ni_*x*_ and Relative Phase Abundance, Obtained from Rietveld Refinement

**sample**		**TiFe *a* [Å]**	**TiFe** [wt%]	**Ti**_**4**_**Fe**_**2**_**O** [wt%]
Ni_0_	TiFe	2.978(3)	98.6(5)	1.4(5)
Ni_5_	TiFe_0.95_Ni_0.05_	2.981(3)	99.6(5)	0.4(5)
Ni_10_	TiFe_0.90_Ni_0.10_	2.982(3)	94.7(5)	5.3(5)
Ni_15_	TiFe_0.85_Ni_0.15_	2.985(3)	97.1(5)	2.9(5)
Ni_20_	TiFe_0.80_Ni_0.20_	2.987(3)	98.9(5)	1.1(5)
Ni_30_	TiFe_0.70_Ni_0.30_	2.993(3)	99.0(5)	1.0(5)

The XRD analyses performed after PCI measurements on hydride samples (see Figure S4) confirmed the structural transformation from a cubic to orthorhombic TiFeH β-phase (space group *P*222_1_).^10,13^ The detection of the suboxide phase in this case was challenging, primarily due to the small molar fraction of Ti_4_Fe_2_O and the overlap of its main Bragg reflections with the broad TiFeH peaks.

### 3.2 Pressure–Composition Isotherms

**Figure 3:**
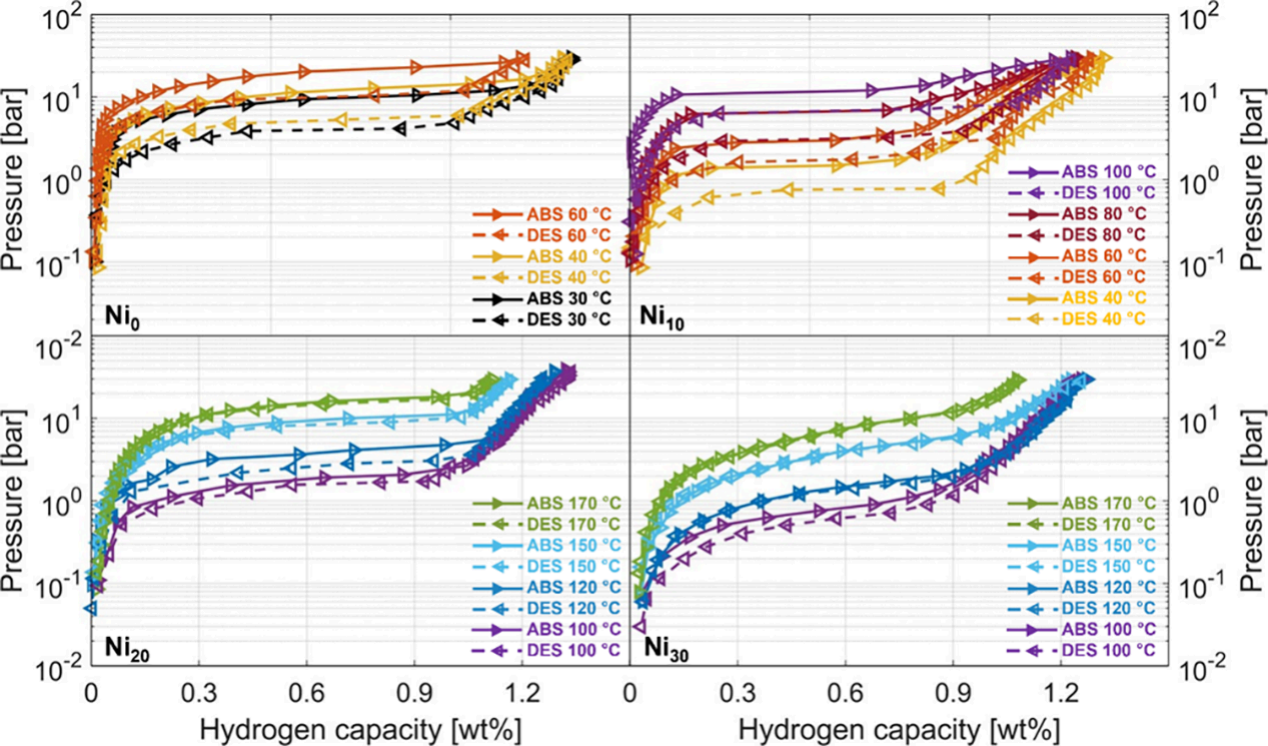
Absorption (right arrows, continuous lines) and desorption (left arrows, broken lines) PCIs for Ni_0_, Ni_10_, Ni_20_, and Ni_30_ in the temperature range 30–170 °C. Error bars within data points. Capacity, hysteresis, and thermodynamic parameters are reported in Tables S1 and S2.

The PCIs for samples Ni_0_, Ni_10_, Ni_20_, and Ni_30_ are shown in Figure 3. Within the investigated pressure range, all curves are characterized by a single plateau corresponding to the formation and decomposition of the monohydride β-phase. Above *H*/*M* ≈ 1, an almost linear trend arises, corresponding to the beginning of the transition to the dihydride γ-phase, which proceeds continuously with the hydrogen content, as reported in the literature.^11^

As shown in Table S1, the addition of Ni does not significantly affect the gravimetric capacity: only a slight reduction, often within the experimental uncertainty, is observed when transitioning from pure TiFe to Ni-substituted alloys with no clear correlation between storage capacity and the Ni content. Specifically, Ni_0_ —that has been tested up to 60 °C because of the high equilibrium pressure reached above that temperature— exhibits a maximum gravimetric density of 1.34(3) wt% at 30 °C and 30 bar. In the same pressure conditions, at 100 °C, Ni_10_ achieves a total capacity of 1.23(3) wt%, while Ni_20_ displays a slightly higher capacity of 1.32(3) wt%, which drops down to 1.26(3) wt% for Ni_30_.

On the other hand, Ni substitution has a pronounced effect on the pressure–temperature operational window because it stabilizes the monohydride reducing its plateau pressure compared to equiatomic TiFe. Figure 3 shows that the absorption plateau pressure at 40 °C decreases from 12.8 bar of Ni_0_ to 1.5 bar of Ni_10_, with a further reduction in Ni_20_ and Ni_30_ even at higher temperatures. Samples with a high Ni content are so stable that it is not possible to determine their full PCIs below 80 °C because some equilibrium points are below the reliable range of the pressure sensor. The consequence of the increased stability is that, considering operation in the 1–30 bar pressure range— typical of storage systems fed by a PEM electrolyzer and coupled with a fuel cell— the working temperatures and reversible gravimetric densities vary substantially with the Ni content. For instance, Ni_30_ can release only half of its reversible capacity at 1 bar unless the temperature exceeds 100 °C. Ni_10_ and Ni_20_ achieve reversible capacities between 1.2 and 1.3 wt% even at 100 °C, where pure TiFe would require much higher absorption pressure. The highest reversible capacities achievable in the 1–30 bar pressure window, and the respective operational temperatures determined from PCIs, are listed in Table S1. The detailed *p*–*T* map reported in Table S2 for absorption and desorption allows calculation of the maximum reversible capacity for different pressure ranges, which may be of interest for specific applications.

Increasing the Ni concentration results in a more sloped plateau. This effect may be ascribed to small fluctuations of the Ni content at the nanoscale, which correspond to a distribution of local compositions. Indeed, inhomogeneities in the chemical environment are typically responsible for the plateau sloping effect,^32,33^ as they can vary hydrogen solubility or introduce chemical potential gradients. Furthermore, we notice that pressure hysteresis decreases with increasing both the temperature and Ni content. It is generally accepted that hysteresis originates from plastic deformation caused by the production and annihilation of dislocations.^34,35^ The hysteresis tends to decrease with increasing temperature because dislocation motion is activated at a smaller level of stress, resulting in a smaller plastic work. Inspection of Figure 3 reveals that increasing the Ni content reduces the hysteresis at constant temperature (compare for instance data at 100 °C for Ni_10_ and Ni_20_). The atomistic mechanism behind the impact of Ni on plastic deformation mechanisms and hysteresis is beyond the scope of this work.

In general, the obtained thermodynamic data are in good agreement with the ones reported in previous works.^21,36−38^

### 3.3 High-Pressure DSC

**Figure 4:**
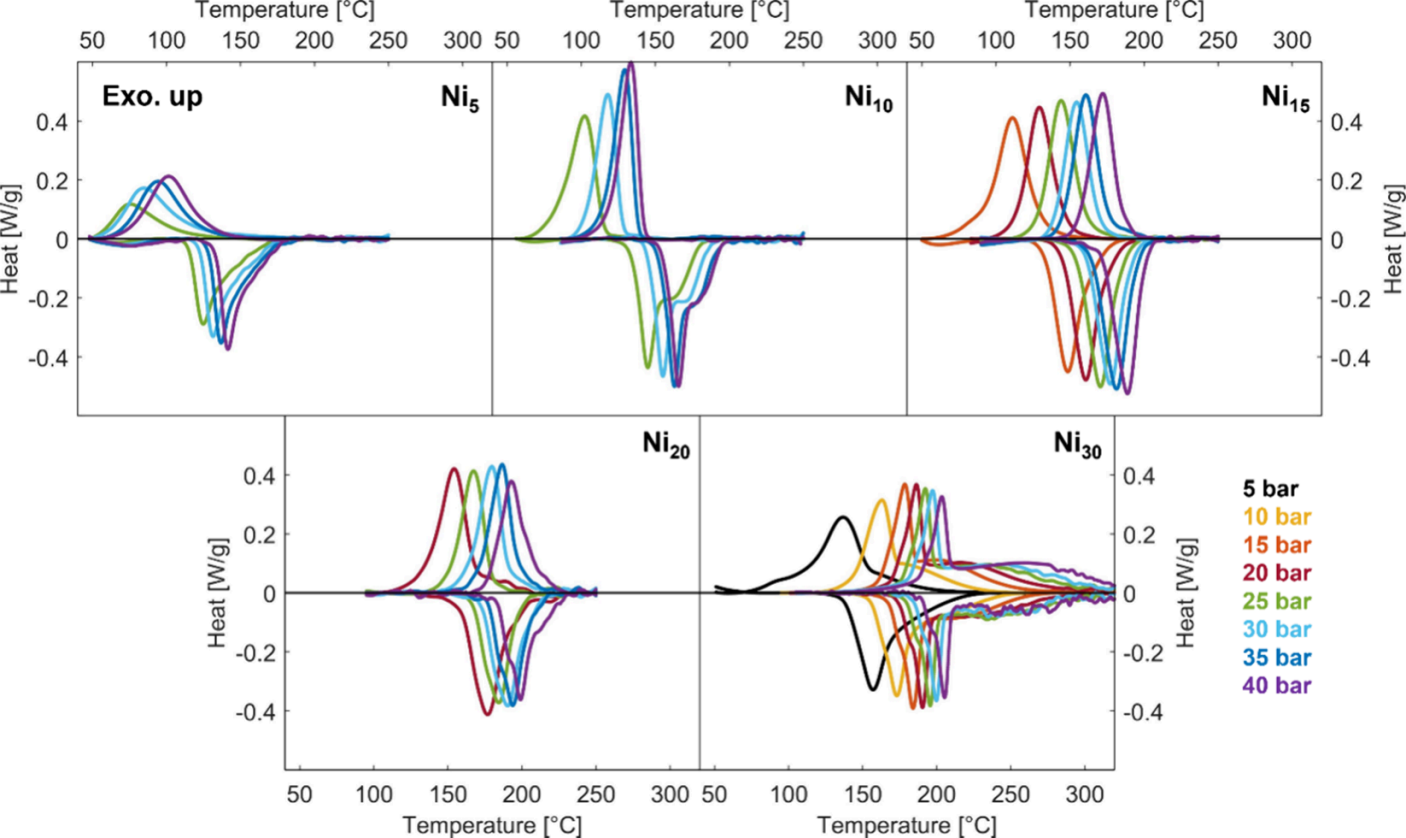
HP-DSC scans of samples Ni_5_ to Ni_30_ (exothermic signal up) at 5 bar (black), 10 bar (yellow), 15 bar (orange), 20 bar (red), 25 bar (green), 30 bar (sky), 35 bar (blue), and 40 bar (violet).

Figure 4 shows the calorimetric profiles for samples Ni_5_ to Ni_30_ (exothermic signal up). From the PCI characterization, we know that in the explored p–T window, the high-pressure transition to the dihydride does not occur or is just in its initial phase. Therefore, the high-temperature shoulders visible in some HP-DSC peaks do not correspond to a second process related to dihydride decomposition (heating) and formation (cooling) because if this was the case, then they should occur at the low-temperature side, given the lower stability of the dihydride. We suggest that the observed shoulders stem from different kinetics in the initial and final part of the transformation due for instance to a transition from a nucleation-limited to a diffusion-limited process or from the presence of a multimodal distribution of powder particle sizes. In particular, Ni_30_ displays a broad shoulder for temperatures above the main peak.

**Figure 5:**
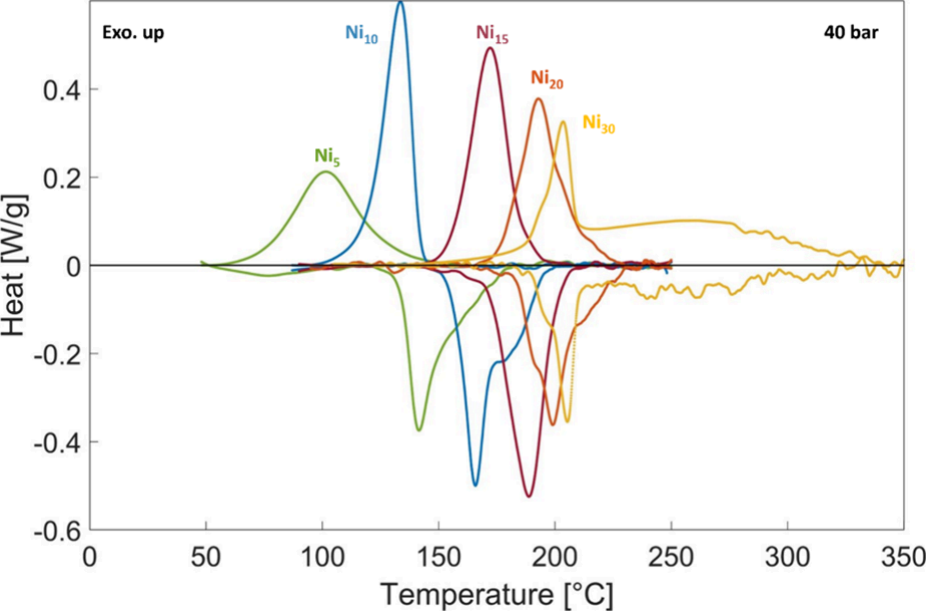
Calorimetric signals of samples Ni_5_ to Ni_30_ at 40 bar (exothermic signal up).

The effect of a larger Ni content is evident in the shift of the calorimetric peaks at 40 bar (Figure 5). As the Ni concentration increases, the decomposition temperature rises, confirming that Ni enhances the stability of the hydride. The mean enthalpy and entropy obtained according to the procedure outlined in Section 2 are reported in Table 3. They show a clear dependence on composition, increasing in absolute value with an increasing Ni content.

**Table 3:** Absolute Value of Enthalpy and Entropy of Hydride Formation^a^

**sample**	**Δ***H*_**DSC**_[kJ mol^–1^]	**Δ***S*_**DSC**_[J K^–1^ mol^–1^]	**Δ***H*_**PCI**_[kJ mol^–1^]	**Δ***S*_**PCI**_[J K^–1^ mol^–1^]
Ni_0_			26.7(1)	103.1(2)
Ni_5_	25.2(8)	94(2)		
Ni_10_	28(1)	98(2)	34.4(1)	110.8(4)
Ni_15_	34.7(3)	107.9(8)		
Ni_20_	39.4(5)	115(1)	43(1)	120(3)
Ni_30_	43(1)	118(3)	45.8(1)	120.0(3)

a See the text for details of the analysis procedure. The second and third columns report the HP-DSC results on all samples from Ni_5_ to Ni_30_; the last two columns report the PCI results on four selected samples. The numbers in parentheses represent the standard error obtained from Van ‘t Hoff fits in units of the last significant digit.

The temperature hysteresis tends to decrease with increasing both the temperature and Ni content, a behavior similar to the one observed in PCIs for the pressure hysteresis.

## 4 Discussion

### 4.1 Effect of Ni on the Lattice Parameter and Thermodynamics

The correlation between the hydride structure and stability has long been debated. Notably, Lundin et al.^27,39^ proved the existence of a strong connection between the tetragonal interstitial size in AB and AB_5_ hydrides and their standard free energy of formation. In their investigation, they observed an abnormal increase in the lattice parameters of TiCo (2.991 Å) and TiNi (3.015 Å) compared to TiFe (2.976 Å), although the atomic radii of Co (1.25 Å)^40^ and Ni (1.24 Å)^25^ are similar to the one of Fe (1.26 Å). This effect has been explained by variations in the bond strength among these compounds. In TiFe, a quasi-ionic bond forms due to increased electron exchange, making it stronger and tighter. In contrast, the bonds in TiNi and TiCo are weaker, resulting in larger lattice parameters, bigger interstitial sizes, and more stable hydrides.^27,39^

In a recent study, Witman et al.^41^ emphasized the importance of improving the predictability of hydride stability using structural insights derived from machine learning (ML) descriptors, such as the specific volume per atom (ν_pa_^Magpie^) for a given composition: ν_pa_^Magpie^ = ∑_*i*_^*N*^*f*_*i*_ν_*i*_ where *f*_*i*_ is the atomic fraction of element *i* and ν_*i*_ is the atomic volume in its ground-state elemental solid form. All the AB_5_ compositions in their analyzed data set showed consistent behavior, aligning with the prediction that a larger ν_pa_^Magpie^ corresponds to a lower plateau pressure in the respective hydride. In our scenario, Ni has a significantly smaller molar volume than Fe (6.59 and 7.09 cm^3^/mol, respectively), resulting in a reduced ν_pa_^Magpie^, decreasing from 8.91/N_A_ cm^3^/atom for Ni_0_ to 8.83/N_A_ cm^3^/atom for Ni_30_ (the molar volume of Ti being 10.64 cm^3^/mol and the elemental fractions corresponding to the compositions measured with EDS). Nonetheless, hydride stabilization is still observed upon Ni incorporation due to the above-mentioned unit cell expansion.

Interestingly, Cuevas et al.^42^ observed a 35% increase in the enthalpy of formation of the investigated AB_5_ hydride due to a 4% expansion in the unit cell volume caused by the incorporation of substituents such as Mn, Al, and Co. In comparison, a 2% enlargement in the Ni-substituted TiFe alloys analyzed in this work resulted in a 60% increase in the stability. This difference can be explained by considering that Cuevas et al. attributed the enhanced stability primarily to the geometric expansion of the cell. In contrast, in our system, incorporating Ni, which shows greater hydrogen affinity compared to Fe, may contribute to stabilizing the hydride to more negative enthalpy values.

We conclude that although ν_pa_^Magpie^ is a powerful parameter derived solely from the hydride composition, it is not a universally significant descriptor. This limitation may arise from its inability to account for the local atomic environment, which is fundamental to fully understanding the behavior of a specific material, as demonstrated by our TiFe_1–*x*_Ni_*x*_. Additional elemental parameters, such as the atomic radius, or structural descriptors, like bond lengths and Vegard’s law coefficient for the variation of the unit cell volume, could provide a more comprehensive framework to better capture the thermodynamics of hydride formation and decomposition.

### 4.2 Enthalpy–Entropy Compensation

**Figure 6:**
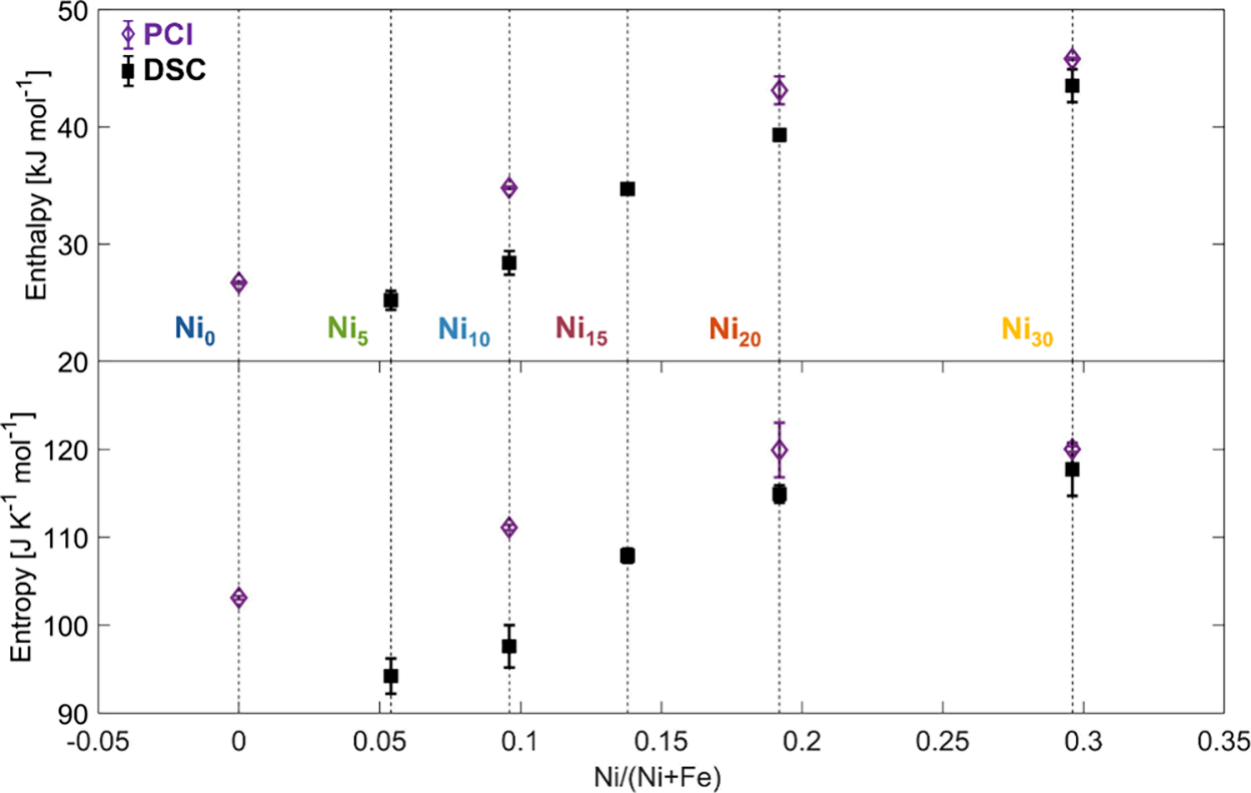
Absolute value of enthalpy and entropy obtained from Van ‘t Hoff analysis of HP-DSC (black squares) and PCI (violet diamonds) data as a function of the Ni content.

As shown in Figure 6 and Table 3, the structural modifications induced by Ni substitution, primarily the expansion of the unit cell as the Ni content increases, significantly affect the thermodynamics of hydride formation and decomposition. These changes lead to higher absolute values of both enthalpies and entropies, resulting in more stable hydrides. The variation is more pronounced at a low Ni content and tends to flatten when going from Ni_20_ to Ni_30_.

**Figure 7:**
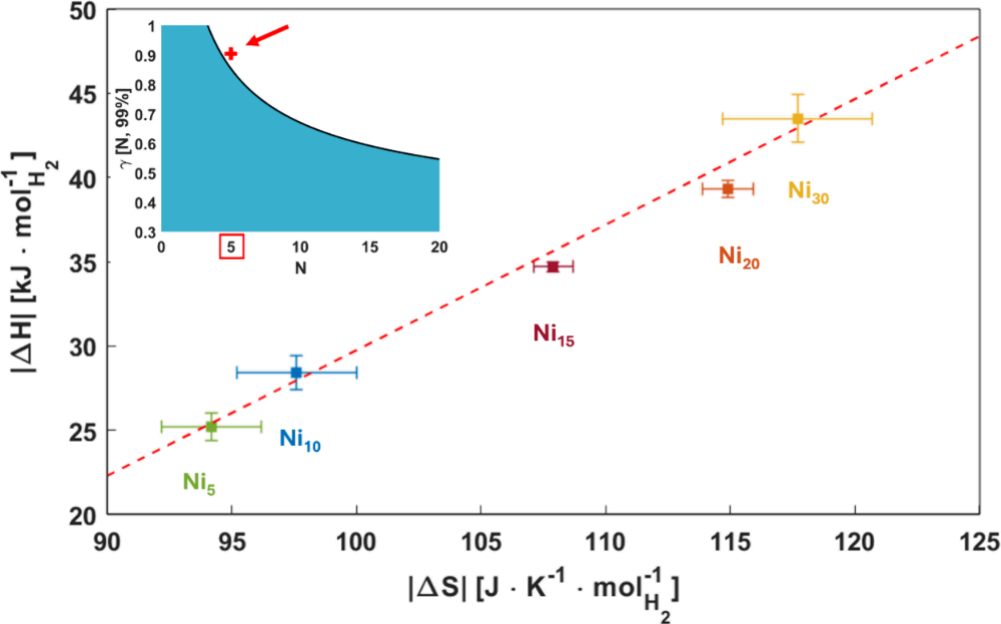
Linear correlation between absolute values of enthalpy and entropy extracted from the Van ‘t Hoff analysis of HP-DSC data. The inset shows the CQF of our data (red cross) obtained following Griessen’s approach^24^ and the threshold γ (black line) for a c.l. of 99%.

**Figure 8:**
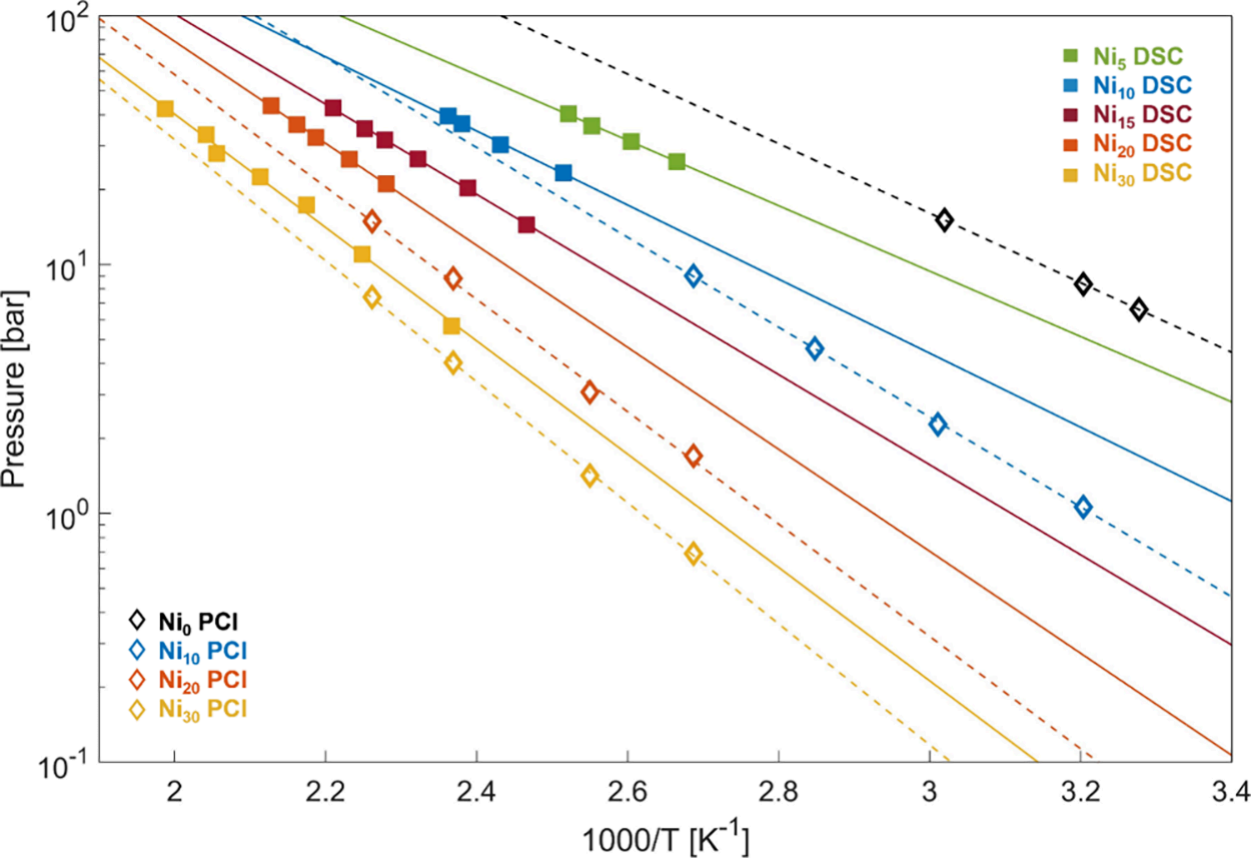
HP-DSC (full squares) and PCI (empty diamonds) mean equilibrium pressure points vs ^–1^. Continuous and broken lines represent the Van ‘t Hoff fit on the collected data. The equilibrium pressure is reduced with an increasing Ni content. There is a 10% underestimation of DSC thermodynamic parameters compared with PCI data.

Interestingly, a linear correlation exists between the enthalpy and entropy values determined for different compositions, as shown in Figure 7 for the data derived by HP-DSC, which is also consistent with the trend shown by the results of PCI analysis (Van ‘t Hoff plots in Figure 8). The apparent enthalpy–entropy compensation (EEC) observed here is often encountered in chemistry, biology, and physics. However, it is often a matter of debate whether it is a genuine effect or a statistical artifact, especially when a narrow temperature range is explored.^43^

In a recent study, Griessen et al.^24,43^ proposed an approach to determine whether the observed EEC arises from statistical artifacts or is a genuine characteristic of the investigated system. In contrast to the findings of Krug et al.,^44,45^ who suggested comparing the compensation temperature (*T*_comp_) obtained from the Δ*H* vs Δ*S* linear fit with the harmonic temperature (*T*_harm_) of the collected data, Griessen et al. introduced a compensation quality factor (CQF) to account for the coalescence of the Van ‘t Hoff plots, enabling a more thorough examination of the compensation phenomenon.

By selecting a threshold value, γ[*N*; c.l.], which depends solely on the number of investigated samples (*N*) and the chosen confidence level (c.l.), it is possible to determine whether the observed EEC is due to a statistical fit compensation (i.e., a limited experimented temperature range) or if it is intrinsic to the system, within the specified confidence level.

In our case, as shown in the SI, *T*_comp_ = 745.7 K, and considering *N* = 5 and a 99% confidence level, we find that γ = 0.85, a smaller value than the CQF determined from our data (CQF = 0.90, see the inset of Figure 7). Therefore, we can conclude with 99% confidence that the EEC effect is genuine and intrinsic to the system.

This leads to the conclusion that the increasing Ni concentration in the intermetallic phase shifts the hydride formation entropy toward larger absolute values, bringing it closer to the standard molar entropy of gaseous H_2_ (130.77 J K^–1^ mol^–1^H_2_).

This effect could be related to a reduction in the configurational entropy contribution^46^ caused by the different availability of interstitial sites per atom.^47^ The shift toward more negative entropy values indicates that Ni-rich hydrides have lower entropy. A possible reason for this is that Ni modifies the local environment inducing a blocking effect that prevents the hydrogen atoms to randomly accommodate in all interstitial sites.^48−50^

Another possibility is that the vibrational entropy, especially the one associated with light H atoms, is lower when Ni replaces Fe. The separation of these effects and the quantification of their relative contributions is not an easy task, from both the experimental and theoretical points of view. However, a deeper understanding of this topic promises, as a reward, a greater degree of control over tailoring thermodynamic properties through elemental substitution.

Revealing the presence of EEC and assessing its entity are crucial for accurately estimating the hydride performance, as the interplay between reaction enthalpy and entropy significantly influences the outcomes. Furthermore, this understanding provides insights into the relationship between composition and thermodynamics in materials where this effect occurs, enabling predictions for similar materials by using computational tools such as machine learning.

### 4.3 Comparison between Results of PCIs and HP-DSC

To explore the thermodynamics of hydride formation in TiFe_1–*x*_Ni_*x*_, the current study combined the analysis of equilibrium PCIs with nonequilibrium HP-DSC scans, with the aim to establish a relationship between the enthalpy and entropy of formation and decomposition and the structural changes brought by Ni incorporation.

The comparison between the two methods is summarized in Table 3 and Figure 6, while in Figure 8, we report the compilation of all Van ’t Hoff plots. We can summarize by saying that HP-DSC analysis tends to underestimate both the enthalpy and entropy compared to the PCI. The relative discrepancy is about 15% in the worst case (Ni_10_) and about 5% in the best case (Ni_30_). This discrepancy arises from a nonaccurate determination of the equilibrium p–T couples, which is unavoidable in nonequilibrium methods. Nevertheless, the qualitative assessment of the dependence of enthalpy and entropy on composition is correctly determined by the proposed HP-DSC peak analysis. This makes HP-DSC a valuable tool for the rapid screening of the effects of elemental substitution for a large number of compositions.

## 5 Conclusions

The general purpose of the present work was to investigate the correlation between Ni-induced structural changes and hydride sorption thermodynamics in TiFe_1–*x*_Ni_*x*_, using two complementary hydrogen sorption characterization techniques: volumetric measurements with a Sieverts’ apparatus and calorimetric measurements with a high-pressure DSC.

- Six compositions (*x* = 0, 0.05, 0.10, 0.15, 0.20, and 0.30) were synthesized with an arc melter, obtaining highly homogeneous and ordered BCC B2 structures, characterized by a uniform TiFe phase with a minor amount (<5 wt%) of Ti_4_Fe_2_O at the surface.
- The lattice parameter of the BCC B2 phase exhibits a linear increase with an increasing Ni content. Ni substitution results in a slight change in the total gravimetric capacity associated with monohydride formation while significantly impacting the hysteresis and slope of the plateau pressures. The most visible effect of Ni incorporation lies in the hydride stabilization, which results in a remarkable reduction of the equilibrium pressure, as shown in the PCIs. The correlation between the unit cell volume and hydride stability is qualitatively similar to the one reported for AB_5_ compounds; however, the expanded volume does not stem from the larger atomic size of the B substituent, but from the weaker bonding with the A-site atom.
- Sieverts’ characterization can be coupled with faster HP-DSC analysis, which gives a relative accuracy of about 10% in the obtained enthalpy–entropy values and a correct qualitative assessment of compositional trends.
- A genuine enthalpy–entropy compensation effect was observed as a function of the Ni content with a confidence level of 99%.

Replacing Fe with Ni allows for tailoring the practical conditions for reversible hydrogen sorption, thus matching the requirements of specific applications. The need for higher stability (i.e., a lower plateau pressure) may arise for several reasons. For example, the storage system may be installed in a location where the average temperature is well above 25 °C, rising the plateau pressure of pure TiFe above 30 bar. A hydride more stable than TiFeH, Ni_10_ or Ni_20_ depending on the operational temperature range, would then be needed to be compliant with the 30 bar output of a PEM electrolyzer. There are also electrolyzers that operate at a pressure <30 bar^12^ that would require coupling with a more stable hydride than TiFeH. Another interesting example is the storage of hydrogen produced by a photoelectrochemical reactor, which can only operate at close to ambient pressure, due to the presence of thin transparent windows. In this case, the coupling to a TiFe_1–*x*_Ni_*x*_ alloy with a carefully selected Ni content appears to be the best option for long-term, footprint, and intrinsically safe hydrogen storage.

## Supporting Information Available

SE-SEM images of polished cross sections for all characterized samples, powder XRD profiles of all samples before and after four PCI cycles, tables containing PCI descriptors (α-phase capacity, reversible capacity in the 1–30 bar range, and hysteresis factor) and thermodynamic parameters (Δ*H* and Δ*S* for absorption and desorption processes), and paragraph containing more details on the theoretical framework of the enthalpy–entropy compensation effect and the calculation chain followed in the present work (PDF)
