# Supplementary material for: Tuning TiFe1–xNix Hydride Thermodynamics through Compositional Tailoring
Source: ACS Appl Energy Mater. 2025 Feb 6;8(4):2135–44. doi: 10.1021/acsaem.4c02625 (PMC11863293; doi:10.1021/acsaem.4c02625)
Supplement: Supplementary file 1 — ae4c02625_si_001.pdf [file ae4c02625_si_001.pdf]

Supporting Information

Tuning $TiFe_{1-x}Ni_x$ hydride thermodynamics through compositional tailoring

Evans Pericoli, Viola Ferretti, Dario Verna and Luca Pasquini*

Department of Physics and Astronomy A. Righi, University of Bologna, Bologna, 40127, Italy

E-mail: evans.pericoli2@unibo.it

S1. SEM

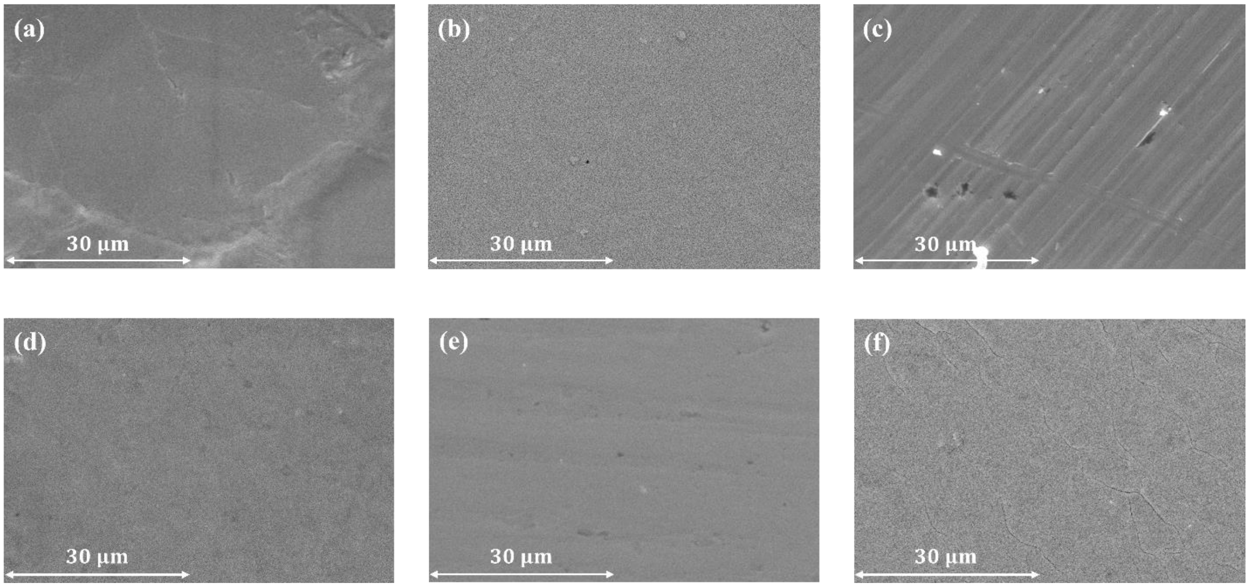

Figure S1: SEM-SE morphology of polished cross sections of samples (a) Ni_0, (b) Ni_5, (c) Ni_{10}, (d) Ni_{15}, (e) Ni_{20}, and (f) Ni_{30} . Local EDS microanalysis on the darker and lighter regions reveal that the composition is the same as in the matrix, indicating that the observed contrast is purely morphological. Precipitates with local composition significantly different from the nominal $TiFe_{1-x}Ni_x$ were not detected, suggesting that the Ti_4Fe_2O phase weakly visible in XRD profiles (see Figures S2-S3) is preferentially located at the surface of the samples produced by arc melting.

S2. XRD

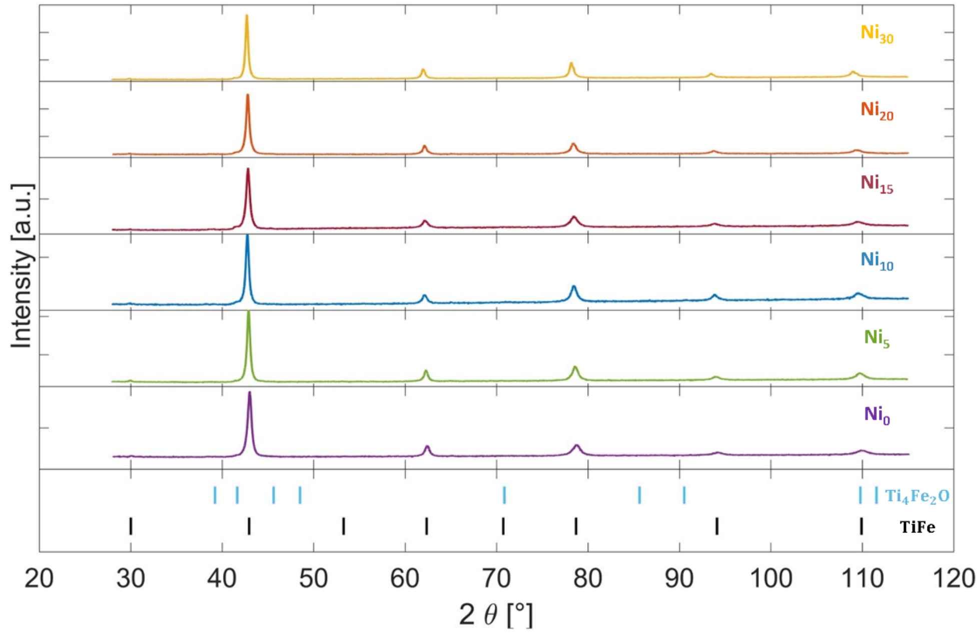

Figure S2: XRD profiles of samples Ni_0 to Ni_{30} after 10 minutes ball milling.

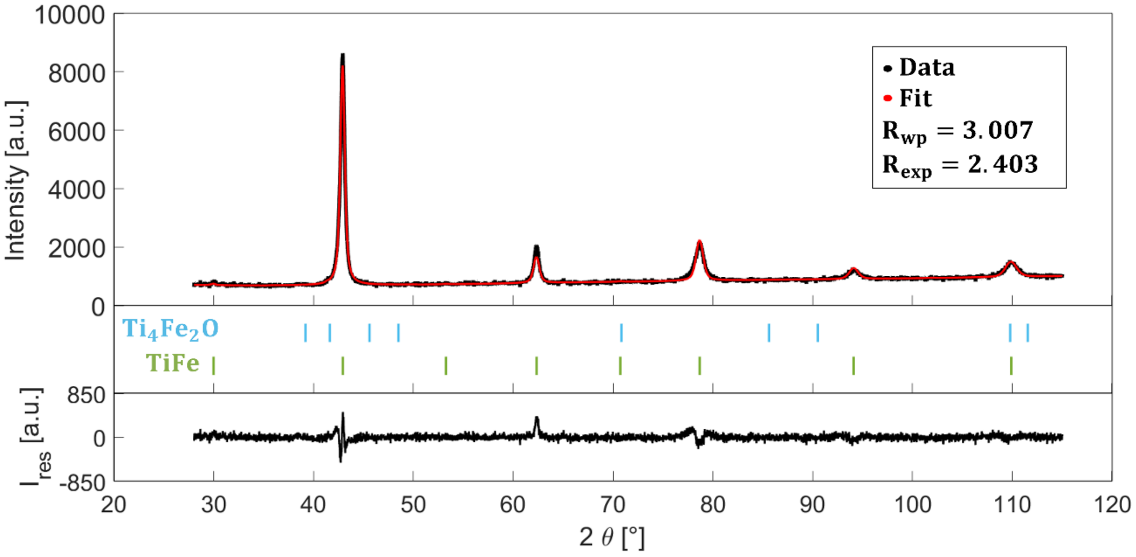

(a)

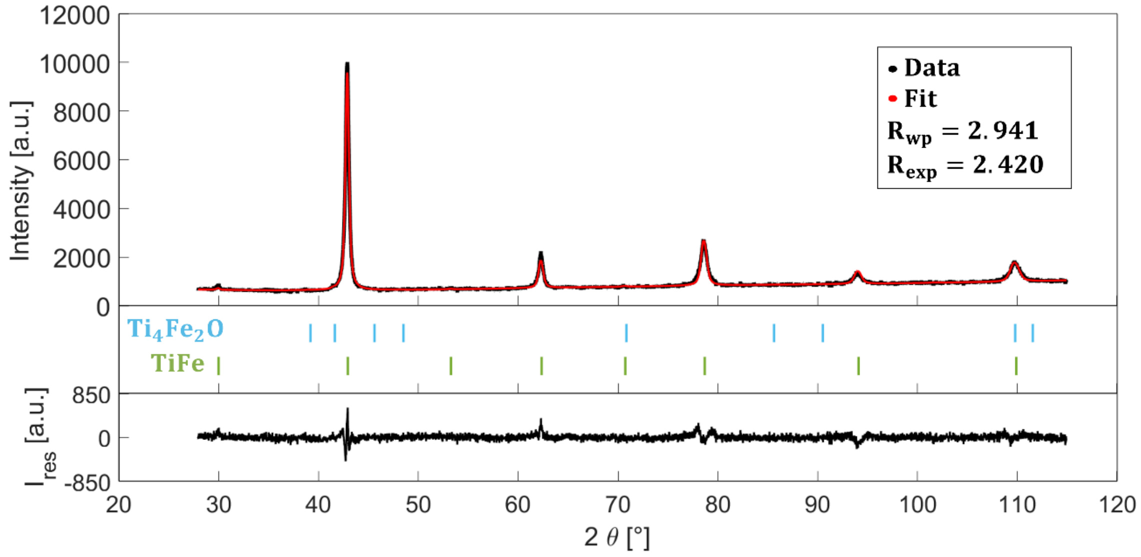
Intensity [a.u.]
Data
Fit
R_{wp} = 2.941
R_{exp} = 2.420
Ti_4Fe_2O
TiFe
I_{res} [a.u.]
2 θ [°]

(b)

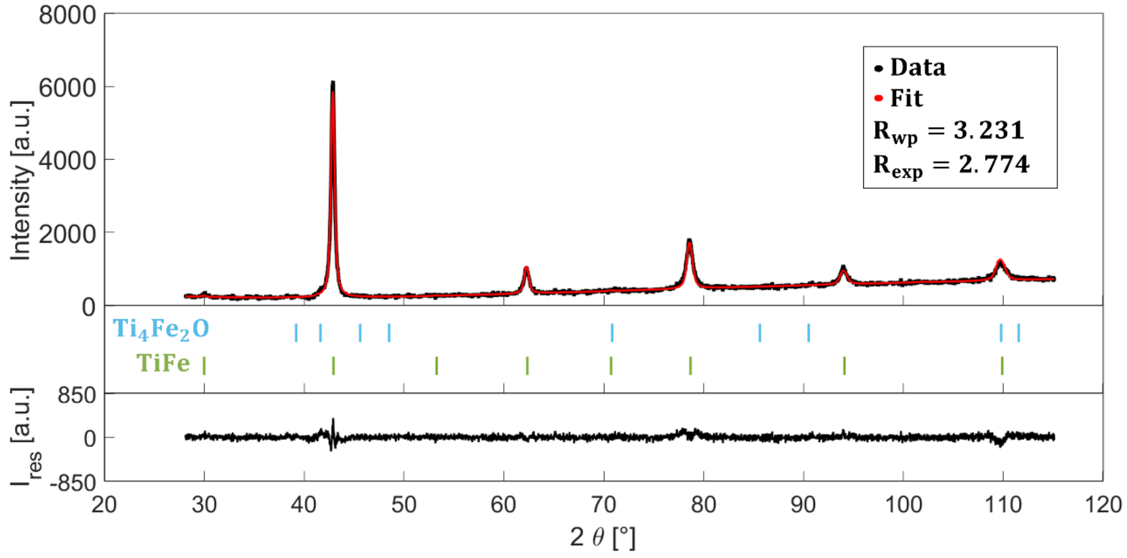
Intensity [a.u.]
Data
Fit
R_{wp} = 3.231
R_{exp} = 2.774
Ti_4Fe_2O
TiFe
I_{res} [a.u.]
2 θ [°]

(c)

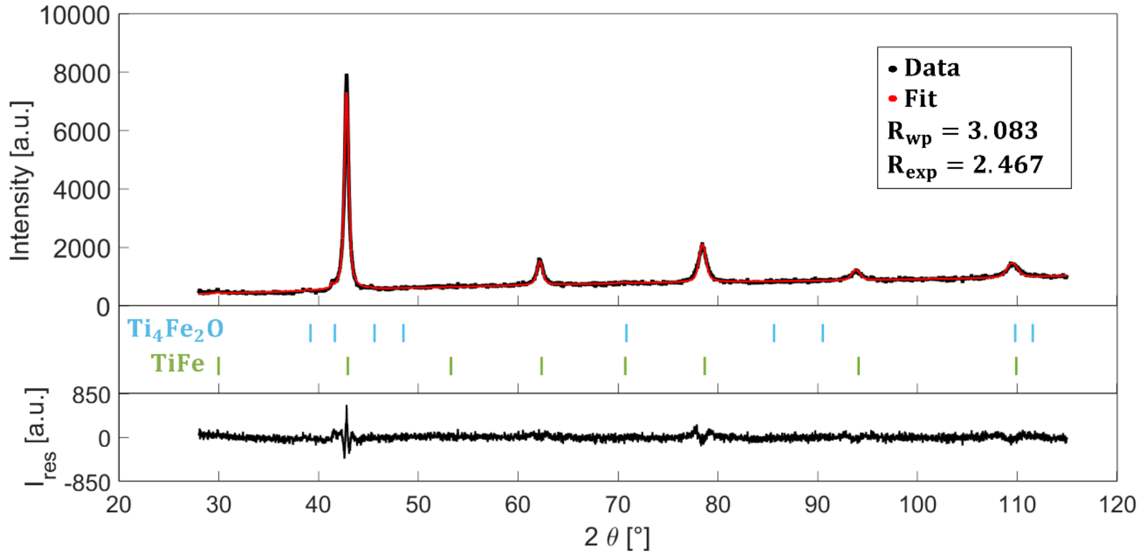
Intensity [a.u.]
Data
Fit
$R_{wp} = 3.083$
$R_{exp} = 2.467$
Ti_4Fe_2O
TiFe
I_{res} [a.u.]
2 θ [°]

(d)

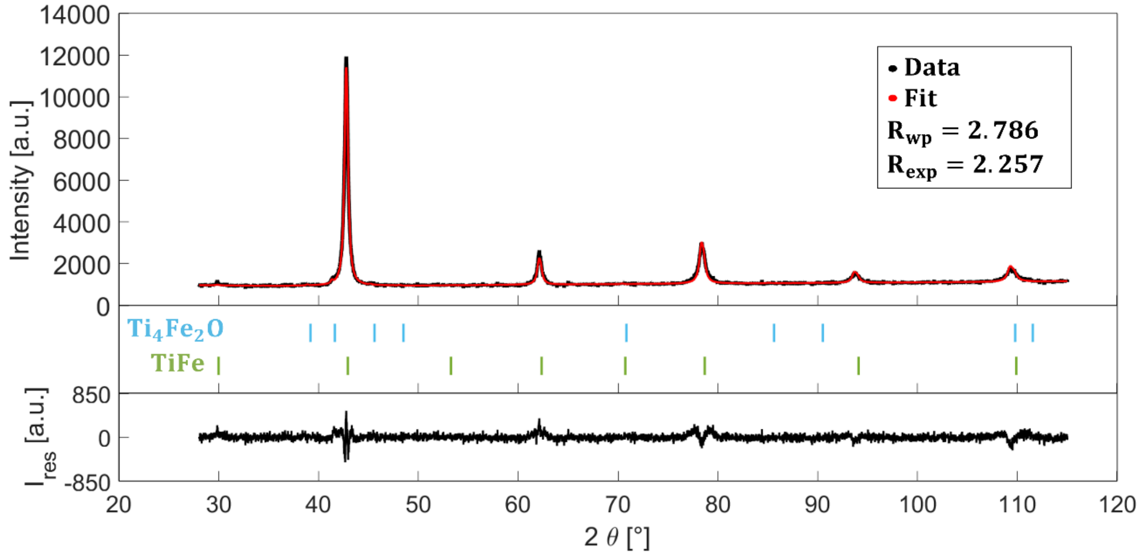
Intensity [a.u.]
Data
Fit
$R_{wp} = 2.786$
$R_{exp} = 2.257$
Ti_4Fe_2O
TiFe
I_{res} [a.u.]
2 θ [°]

(e)

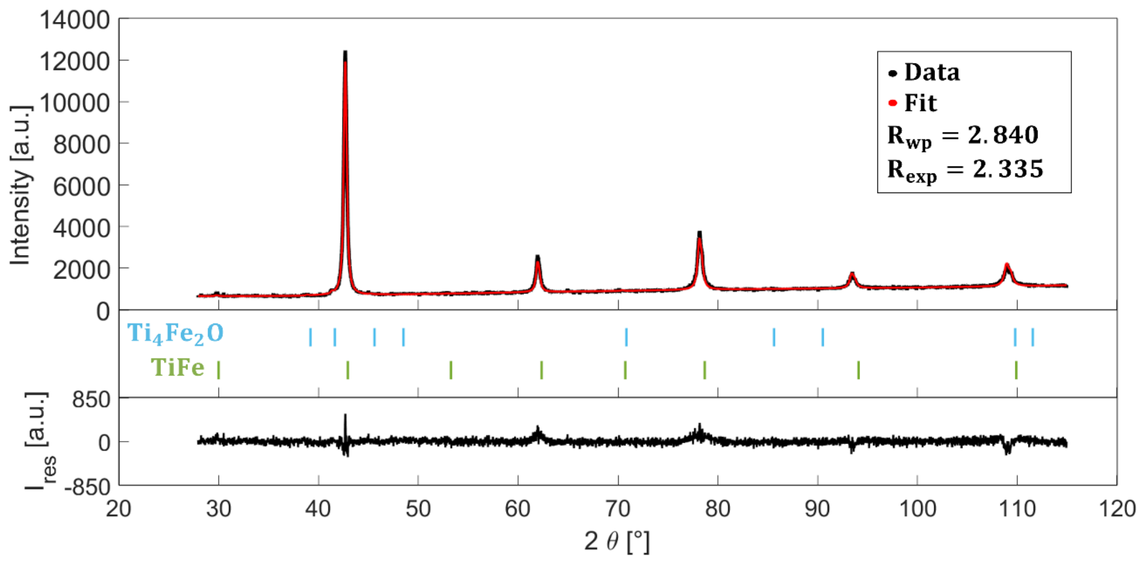

(f)

Figure S3: Rietveld Refinement results for sample (a) Ni_0, (b) Ni_5, (c) Ni_{10}, (d) Ni_{15}, (e) Ni_{20}, and (f) Ni_{30}. The XRD pattern, the fit and the residual plot are reported for each sample, together with R_{wp} and R_{exp} parameters.

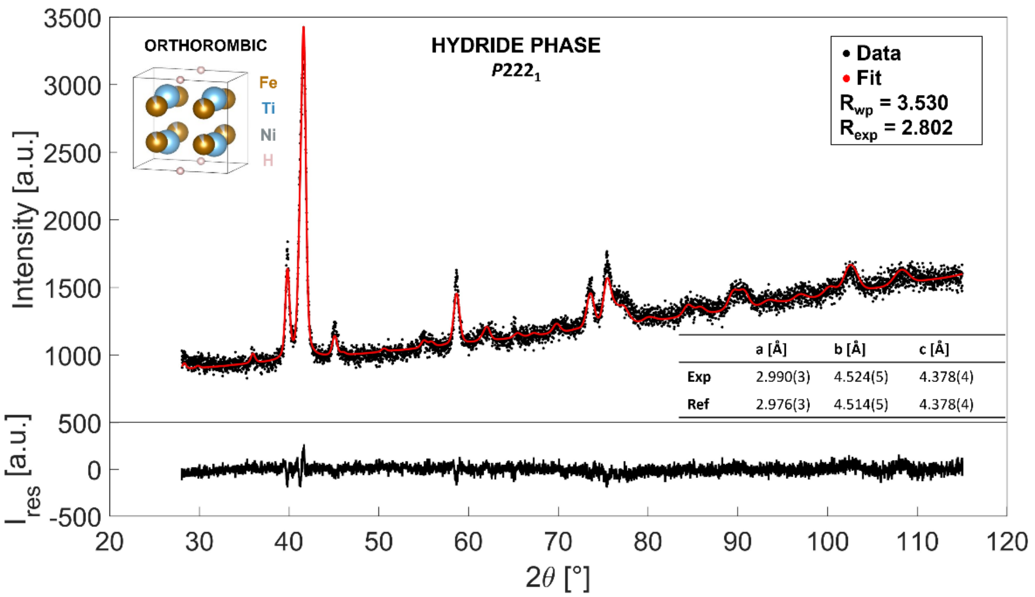

Figure S4: XRD diffractogram and Rietveld refinement of sample Ni_{10} after PCI. The insertion shows the orthorhombic phase of the monohydride. Lattice parameters from the refinement are compared with reference pattern (ICSD code: 00-026-0806).

S3. PCI

Table S1: PCI parameters for samples Ni_0, Ni_{10}, Ni_{20} and Ni_{30}. Total and reversible capacities are reported with the uncertainty in units of the last significant digit and the pressure-temperature conditions of the hydride.

Sample	α phase cap. [wt%]	Tot. Cap. [wt%] 100 °C (30 bar)	Rev. Cap. [wt%] 100 °C (1-30 bar)	$\ln\left(\frac{p_{abs}}{p_{des}}\right)$ (100 °C)
**Ni_0**	0.09	1.34(3) [30°C]	1.28(3) [60°C]	0.74 (60 °C)
**Ni_{10}**	0.12	1.23(3)	1.21(3)	0.61
**Ni_{20}**	0.11	1.32(3)	1.30(3)	0.15
**Ni_{30}**	0.09	1.26(3)	0.48(2)	0.11

Table S2: Absorption and desorption PCI pressure-temperature dataset for sample Ni_0, Ni_{10}, Ni_{20} and Ni_{30}. Enthalpy and entropy are extracted from the Van't Hoff analysis, with the number in parentheses representing the standard error in units of the last significant digit.

**Absorption**										
**Sample**	**Plateau pressure [bar]**								ΔH_{ABS} $[kJ\ mol_{H_2}^{-1}]$	ΔS_{ABS} $[J\ K^{-1} mol_{H_2}^{-1}]$
	**30 °C**	**40 °C**	**60 °C**	**80 °C**	**100 °C**	**120 °C**	**150 °C**	**170 °C**		
**Ni_0**	10.52	12.75	21.90	-	-	-	-	-	-23.8(6)	-98(2)
**Ni_{10}**	-	1.47	2.97	6.60	11.8	-	-	-	-34(1)	-112(3)
**Ni_{20}**	-	-	-	-	1.84	3.65	9.35	15.40	-41.6(6)	-117(1)
**Ni_{30}**	-	-	-	-	0.80	1.50	4.05	7.60	-43.5(9)	-115(2)

**Desorption**										
**Sample**	**Plateau pressure [bar]**								ΔH_{DES} $[kJ\ mol_{H_2}^{-1}]$	ΔS_{DES} $[J\ K^{-1}\ mol_{H_2}^{-1}]$
	**30 °C**	**40 °C**	**60 °C**	**80 °C**	**100 °C**	**120 °C**	**150 °C**	**170 °C**		
**Ni_0**	4.15	5.47	10.38	-	-	-	-	-	29.5(5)	109(2)
**Ni_{10}**	-	0.76	1.75	3.2	6.9	-	-	-	35(1)	110(4)
**Ni_{20}**	-	-	-	-	1.58	2.58	8.30	14.45	44(3)	122(7)
**Ni_{30}**	-	-	-	-	0.60	1.35	4.00	7.20	48(1)	125(3)

S4. HP-DSC

Table S3: Samples equilibrium pressures and temperatures from DSC experiments.

Sample	T [°C]	P [bar]
**Ni_5**	123.4	40.3
	118.6	36.1
	110.8	31.3
	102.1	25.9
**Ni_{10}**	150.2	39.5
	146.9	36.8
	138.1	30.4
	124.4	23.4
**Ni_{15}**	179.4	42.6
	170.9	35.2
	165.8	31.6
	157.4	26.6
	145.5	20.3
	132.4	14.5
**Ni_{20}**	196.8	43.5
	189.4	36.6
	184.1	32.4
	175.0	26.5
	165.4	21.1
**Ni_{30}**	230.0	42.3
	216.6	33.2
	213.3	28.0
	200.0	22.5
	186.7	17.4
	171.6	11.0
	149.4	5.7

S5. EEC

The resulting thermodynamic parameters from Van 't Hoff analysis on DSC data clearly show a simultaneous increase of both the enthalpy (ΔH) and the entropy (ΔS) of reaction with increasing Ni fraction, which can be fitted to a linear relation of the form:

$$\Delta H = a\Delta S + bR$$

Where the angular coefficient is

$$a = T_{comp} = \frac{d(\Delta H)}{d(\Delta S)}$$

From our HP-DSC data $T_{comp} = 745.7K$.

The EEC can in some cases be an artefact, due for instance to the limited data statistics in the p-T plane, forcing Van 't Hoff plots to encounter in an intersection point. An indication of the EEC genuinity is often the agreement between T_{comp} and the harmonic mean temperature $T_{harm} = \left(\frac{1}{M}\sum_i^M \frac{1}{T_i}\right)^{-1}$. However, this does not consider the degree of coalescence among Van 't Hoff fits along the axis. The more recent model developed by Griessen *et al.*[1] considers this aspect, which can lead to a more complete analysis on the p-T plane.

The function to study is the variance $\sigma^2(T)$ of $\ln(p)$, which measures the spread among the Van 't Hoff fits related to different samples, depending on the temperature. Two parameters are of interest:

- T_{min}: the temperature at which the spread $\sigma^2(T)$ is minimum
- T_{max}: the temperature at which $\sigma^2(T)$ is maximum

T_{min} is intuitively in the coalescence region of the Van 't Hoff fits related to the different samples. By minimizing $\sigma^2(T)$, one finds:

$$T_{min} = \frac{T_{comp}}{R^2}$$

Where R^2 is obtained by the linear fit of ΔH vs ΔS.

From our HP-DSC data: $R^2 = 0.9907$, and $T_{min} = 752.7\ K$

On the other side, T_{max} is the point of maximum $\sigma^2(T)$ and is given by the minimum temperature present in the dataset of equilibrium (p,T) points used for the Van 't Hoff fits, which in our case is $T_{max} = 375.2\ K$ (see **Table S3**).

In the end, these two parameters are used to compute the Compensation Quality Factor:

$$CQF = 1 - \sqrt{\frac{\sigma^2(T_{min})}{\sigma^2(T_{max})}}$$

A larger CQF corresponds to a smaller ratio between $\sigma^2(T_{min})$ and $\sigma^2(T_{max})$, which in turn indicates a wider analyzed region in the p-T plane and minimizes the effect of statistical artefacts. From our HP-DSC data we obtain: $CQF = 0.90$.

Boundary conditions to understand if the EEC is genuine are represented by the function $\gamma(N, c.l.\%)$, defined for N samples and the confidence level $c.l.\%$: if $CQF > \gamma$, EEC is a real effect. For $N \geq 5$, a confidence level of 99% is strict enough to determine the EEC source.

The reference function for boundary condition has been determined by Griessen *et al.* by considering statistically independent randomly generated Van ’t Hoff lines for a set of N samples and evaluating the threshold above the 99% of the randomly generated $CQFs$, identified by the following function:

$$\gamma(N, 99\%) = 0.29 + \frac{1.41}{N^{0.57}}$$

In conclusion, from our DSC data:

$\gamma(N = 5{,}99\%) = 0.85 < CQF = 0.90$

We thus conclude that the EEC effect has a genuine nature.

References

(1) Griessen, R.; Boelsma, C.; Schreuders, H.; Broedersz, C. P.; Gremaud, R.; Dam, B. Single Quality Factor for Enthalpy-Entropy Compensation, Isoequilibrium and Isokinetic Relationships. *ChemPhysChem* **2020**, *21* (15), 1632–1643. https://doi.org/https://doi.org/10.1002/cphc.202000390.
